# Small RNA and Degradome Deep Sequencing Reveals the Roles of microRNAs in Peanut (*Arachis hypogaea* L.) Cold Response

**DOI:** 10.3389/fpls.2022.920195

**Published:** 2022-06-02

**Authors:** Xin Zhang, Chao Ren, Yunyun Xue, Yuexia Tian, Huiqi Zhang, Na Li, Cong Sheng, Huifang Jiang, Dongmei Bai

**Affiliations:** ^1^Institute of Industrial Crops, Shanxi Agricultural University, Taiyuan, China; ^2^State Key Laboratory of Sustainable Dryland Agriculture, Shanxi Agricultural University, Taiyuan, China; ^3^Department of Plant Pathology, College of Plant Protection, Nanjing Agricultural University, Nanjing, China; ^4^Laboratory of Bio-interactions and Crop Health, Nanjing Agricultural University, Nanjing, China; ^5^Key Laboratory of Biology and Genetic Improvement of Oil Crops, Ministry of Agriculture, Oil Crops Research Institute of the Chinese Academy of Agricultural Sciences, Wuhan, China

**Keywords:** peanut, microRNA, cold stress, cold response, cold tolerance

## Abstract

Cold stress is a major environmental factor that affects plant growth and development, as well as fruit postharvest life and quality. MicroRNAs (miRNAs) are a class of non-coding small RNAs that play crucial roles in various abiotic stresses. Peanuts (*Arachis hypogaea* L.), one of the most important grain legumes and source of edible oils and proteins, are cultivated in the semi-arid tropical and subtropical regions of the world. To date, there has been no report on the role of miRNAs in the response to cold stress in cultivated peanuts. In this study, we profiled cold-responsive miRNAs in peanuts using deep sequencing in cold-sensitive (WQL20) alongside a tolerant variety (WQL30). A total of 407 known miRNAs and 143 novel peanut-specific miRNAs were identified. The expression of selected known and novel miRNAs was validated by northern blotting and six known cold-responsive miRNAs were revealed. Degradome sequencing identified six cold-responsive miRNAs that regulate 12 target genes. The correlative expression patterns of several miRNAs and their target genes were further validated using qRT-PCR. Our data showed that miR160-*ARF*, miR482-*WDRL*, miR2118-*DR*, miR396-*GRF*, miR162-*DCL*, miR1511-*SRF,* and miR1511-*SPIRAL1* modules may mediate cold stress responses. Transient expression analysis in *Nicotiana benthamiana* found that miR160, miR482, and miR2118 may play positive roles, and miR396, miR162, and miR1511 play negative roles in the regulation of peanut cold tolerance. Our results provide a foundation for understanding miRNA-dependent cold stress response in peanuts. The characterized correlations between miRNAs and their response to cold stress could serve as markers in breeding programs or tools for improving cold tolerance of peanuts.

## Introduction

Cultivated peanuts (*Arachis hypogaea* L.) are vital oil plants and cash crops that globally play an essential role in consuming edible vegetable oil and leisure food. However, low temperatures limit the scope of crops to a large extent and cause yield reduction, quality decline, and occasionally no harvest in severe cases (Jeon and Kim, 2013; Barrero-Gil and Salinas, 2018). The annual loss of crops caused by low-temperature damage worldwide is as high as hundreds of billions of dollars (Xiao and Song, 2011; Abla et al., 2019). Therefore, the improvement of cold tolerance has essential application value for crops and other plants and is an urgent research topic.

As a thermophilic crop, peanuts require relatively high temperatures throughout their development. Cold injury causes different degrees of harm to the peanut at seedling emergence, flowering, maturity, and other key growth stages. Among them, the impact of the seedling stage is the most common, which could delay peanut germination and seedling growth, resulting in uneven seedling emergence, weak seedling potential, prolonged seedling emergence time, lack of seedlings, and ridge cutting. Research on cold stress in plants began in the early 1830s and today has a history of more than 190 years. Breeders have been trying to develop new varieties to resolve the problem of peanut chilling damage and have made progress. A few cold-tolerant and early maturing varieties have been cultivated (Upadhyaya et al., 2003; Zhang et al., 2019). However, the mechanism of cold tolerance is a complex quantitative trait that always appears in combination or continuously and is not controlled by a single regulatory pathway or gene, making traditional cold tolerance breeding methods challenging with longer breeding cycles (Park et al., 2015; Shi et al., 2018; Zhang et al., 2019). Therefore, understanding peanut cold defense mechanisms is necessary to accelerate the cultivation of peanut varieties with high cold tolerance. Under low non-freezing temperature conditions, plants reprogram their gene expression through transcriptional, post-transcriptional, and post-translational mechanisms and undergo physiological and biochemical adjustments to improve their tolerance to low temperatures. Many studies have shown that the molecular mechanisms of cold defense in many plants are evident. The plant’s cold defense molecular mechanism is a highly complex network composed of numerous positive and negative regulatory factors, which can form a center that integrates a variety of internal and external signals by binding to the C-repeat binding transcription factor (CBF)/dehydrate responsive element binding factor (DREB) promoter (Barrero-Gil and Salinas, 2018). The core mechanism is that cold stress activates the plant’s ICE-CBF-COR regulatory pathway. Under cold stress, the transcription factor CBF responds to cold signals with the participation of the transcription activator ICE (inducer of CBF expression), binds to the cis-acting element CRT (C-repeat)/DER (dehydration) of the cold-induced genes COR (cold-regulated) responsive element sequence, initiates the expression of a series of cold-induced genes and proteins, and then changes the physiology, metabolism, and growth of plants (Gilmour et al., 1998; Wang et al., 2017; Barrero-Gil and Salinas, 2018; Shi et al., 2018; Guo et al., 2019). The ICE-CBF-COR transcriptional cascade has been established as the main regulatory response toolkit for cold signaling and freezing tolerance. Peanuts are sensitive to low temperatures, but they have evolved a unique low-temperature survival mechanism (Zhu, 2016). Studies have reported that overexpression of *AtDREB2A* in peanuts can improve their tolerance to low temperature, drought, and salt stress (Pruthvi et al., 2014). *AhPNDREB1* (a homologous gene of *AtCBF1*), which is intron-less and constitutively expressed, was isolated from the cultivar Luhua 14 (Zhang et al., 2009). The AP2 domain in *AhPNDREB1* is specific to the CRT/DER element combining ability, and the gene can be vigorously and rapidly induced to be expressed under cold and drought stress. Chen et al. (2012) cloned peanut *AhCBF2* and *AhCBF15*, analyzed their amino acid sequences, and found that they both contained the unique ERF/AP2 domain of CBF/DREB family genes. However, there are few reports on the mechanism of peanut cold tolerance, which requires further exploration.

Over the past few years, many studies have shown that small RNA (sRNA) play a pivotal role in regulating cold stress in various plants. sRNA, as a type–21-24 nucleotide long non-coding RNA molecule, is widespread in eukaryotes and has two main forms: microRNA (miRNA) and small interfering RNA (siRNA) (Jin, 2008). It can form an RNA-induced silencing complex by combining with the ribozyme complex containing the AGO protein to cleave target mRNA or inhibit its translation and is widely involved in the cold stress responses of various plants, such as *Arabidopsis* (Sunkar and Zhu, 2004; Zhou et al., 2008; Yan et al. 2016), poplar (Lu et al., 2008), wheat (Tang et al., 2012), rice (Yang et al., 2013), soybean (Zhang et al., 2014), tomato (Shi et al., 2019), and wild grape (Wang et al., 2019). By targeting stress-related transcription factors, such as MYB, WRKY, and bHLH (Zhang et al., 2014; Shi et al., 2019; Wang et al., 2019), genes related to the reactive oxygen scavenging system (Yang et al., 2013) or ABA signaling pathway genes (Yan et al., 2016), these miRNAs may regulate the cold tolerance of plants. Studies have shown that sRNA-mediated gene silencing, histone modification, phosphorylation, and ubiquitination can regulate CBF expression at post-transcriptional and post-translational levels (Barrero-Gil and Salinas, 2018; Lantzouni et al., 2020). Thus, we believe that exploring the peanut cold defense regulatory network from small RNA-mediated gene silencing will be helpful in revealing the peanut response and regulatory mechanisms of cold stress.

To understand the role of miRNAs in peanut cold tolerance, we used two representative peanut lines in this study: WQL20 (waiting for quiz line 20) and WQL30 were eighth-generation recombinant inbred lines (RILs) from a cross between cultivars of Yuanza 9102 (female) and Xuzhou 68–4 (male). The main difference between the two RILs is their ability to withstand low temperatures: WQL30 is resistant to low temperatures, whereas WQL20 is susceptible to low temperatures. These two peanut lines were investigated to understand peanut cold tolerance by analyzing plant miRNA expression patterns and their target genes.

## Materials and Methods

### Plant Materials

The RIL population from a cross between cultivars of Yuanza 9102 (female) and Xuzhou 68–4 (male) includes 188 F_8_ lines. Yuanza 9102, the female parent of the RIL population, belonging to *A. hypogaea* subsp. fastigiata var. vulgaris, was derived from interspecific hybridization between cultivated cultivar Baisha 1,016 and a diploid wild species *A. diogoi*. Xuzhou 68–4, the male parent, which belongs to *A. hypogaea* subsp. hypogaea var. hypogaea. Yuanza 9102 is a popular cultivar in China that is resistant to cold stress, while Xuzhou 68–4 is susceptible to cold stress.

**Figure 1:**
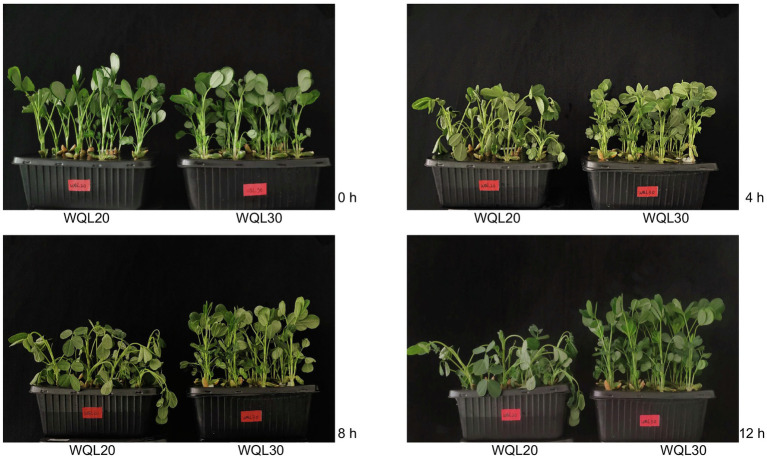
The phenotype of peanut exposed to low temperature. For low-temperature treatment, four-leaf stage seedlings of WQL30 and WQL20 were kept in a growth chamber at 6°C, 70% RH, and a 14-h/10-h light/dark photoperiod. With the increase of treatment time, WQL20 began to wilt, while WQL30 did not change significantly after 4 h of cold treatment. After 8 h of cold treatment, WQL20 wilted significantly, but WQL30 still had no noticeable change. After 12 h of cold treatment, WQL20 wilted and fell, while WQL30 showed slight wilting symptoms as shown in this figure. The pictures were taken at 0 HCT, 4 HCT, 8 HCT, and 12 HCT and assembled in Adobe Photoshop CS6.

We conducted hydroponic culture of the two lines in Hoagland solution in a growth chamber, and then culture them in illumination incubator in the laboratory. The temperature was 27°C and the light–dark cycle was 14 h of light and 10  h of darkness. For low-temperature treatment, 6 four-leaf stage seedlings for each line were kept at 6°C, 70% RH, and a 14-h/10-h light/dark photoperiod. Lines WQL30 and WQL20 showed apparent difference resistance to cold. After 4 h of cold treatment (HCT), WQL20 began to wilt, while WQL30 did not change significantly. After 8 h of cold treatment (HCT), WQL20 wilted significantly, but WQL30 still had no noticeable change. After 12 h of cold treatment (HCT), WQL20 wilted and fell, while WQL30 showed slight wilting symptoms (Figure 1). Samples of four HCT (0 h, 4 h, 8 h, and 12 h) were collected from whole seedlings for miRNAome and degradome analysis.

### sRNA Library Construction and DNA Sequencing

RNA degradation and contamination was monitored on 1% agarose gels. RNA purity was checked using the NanoPhotometer^®^ spectrophotometer (IMPLEN, CA, United States). RNA concentration was measured using Qubit® RNA Assay Kit in Qubit^®^ 2.0 Fluorometer (Life Technologies, CA, United States). A total amount of 3 μg total RNA per sample was used as input material for the small RNA library. Sequencing libraries were generated using NEBNext^®^Multiplex Small RNA Library Prep Set for Illumina^®^ (NEB, United States.) following manufacturer’s recommendations and index codes were added to attribute sequences to each sample. Briefly, Total RNA was ligated to the RNA 3′ and RNA 5′ adapters and reverse transcription, followed by PCR, was performed to make cDNA constructs of the small RNAs. PCR products were purified on a 8% polyacrylamide gel (100 V, 80 min). DNA fragments corresponding to 140 ~ 160 bp (the length of small non-coding RNA plus the 3′ and 5′ adaptors) were recovered and dissolved in 8 μl elution buffer. At last, library quality was assessed on the Agilent Bioanalyzer 2,100 system using DNA High Sensitivity Chips. We then performed single-end sequencing (50 bp) on an Illumina Hiseq2500 at the LC-BIO (Novogene Experimental Department, Beijing, China) following the vendor’s recommended protocol.

### Degradome Library Construction and Sequencing

To identify the potential targets, equal amounts of RNAs from WQL20 (0 HCT, 4 HCT, 8 HCT, and 12 HCT) and WQL30 samples (0 HCT, 4 HCT, 8 HCT, and 12 HCT) were mixed together for degradome library construction and deep sequencing. The two libraries named as DS1 and DS2, respectively. Through preprocessing, clean tags are generated. Then, clean tags were classified by alignment with GenBank, Rfam database, and miRNA database. Next, the reads were mapped to the reference genome.^1^ The sense strand of peanut cDNA was used to predict miRNA cleavage sites using CLeaveL and pipeline (Addo-Quaye et al., 2009). Based on the signature number and abundance of cleaved position at each occupied transcript, the cleaved transcripts could be categorized into five categories (0, 1, 2, 3, and 4).

### Northern Blotting Analysis

RNA blot analyses for miRNAs from total extracts were performed as described previously (Katiyar-Agarwal and Jin, 2007). Total RNA was extracted using TRIzol reagent (Takara, Japan) following the manufacturer’s instructions. RNA was resolved on a 14% denaturing 8 M urea-PAGE gel and then transferred and chemically crosslinked onto a Hybond N^+^ membrane (GE Healthcare Life Sciences) using N-(3-Dimethylaminopropyl)-N′-ethylcarbodiimide hydrochloride. miRNA probes were end-labeled with [r-^32^P] ATP by T4 polynucleotide kinase (New England Biolabs). For internal control experiments, blots were stripped for 20 min and 4 times at 80°C in stripping buffer (0.1 X SSC/0.5% SDS). After detecting no signal, the stripped blots were rehybridized with r-^32^P ATP-labeled U6 gene fragments to confirm loading amounts. All signals were normalized to the U6 signals obtained from each blot. Expression levels were quantified using Image J as instructed.

### qRT-PCR

The RNA used for qRT-PCR was from the same set RNA for northern blotting analysis. The RNA was then reverse transcribed into cDNA using the PrimeScript RT reagent Kit (Takara, Japan). The qRT-PCR was performed in a 15 μl reaction mixture consisting of 1.5 μl 1 × SYBR Green (Invitrogen, United States), 1.5 μl PCR buffer, 0.3 μl 10 mM dNTPs (Takara, Japan), 0.3 μl Taq, 0.3 μl ROX DYE2 (Vazyme, China), 1.5 μl 2 mM each primer and 2 μl appropriate diluted cDNA. The conditions for qRT-PCR were as follow: 94°C for 3 min, then 40 cycles at 94°C for 30 s and 58°C for 30 s, followed by 72°C for 35 s for PCR amplification. Transcript levels of each gene were measured by the Applied Biosystems 7500 (Applied Biosystems, United States) according to the manufacturer’s instructions. 18S rRNA was used as a quantitative control in the qRT-PCR analysis. Primers used in this study are listed in Supplementary Table S4.

### Transient Expression Analysis of miRNA in *Nicotiana benthamiana*

The precursor of miRNA, *MIRNA*, cloned from peanut was sub-cloned into the overexpression vector pCAMBIA1300 destination vector using LR clonase II (Invitrogen). Transient co-expression assays in *N*. *benthamiana* were performed by infiltrating 4-week-old *N*. *benthamiana* plants with Agrobacterium GV3101 [OD600 (optical density at 600 nm) = 1.0] harboring constructs containing the *MIRNA* (pCAMBIA1300). The Agrobacterium-mediated transformation was performed according to a previously described method (Niu et al., 2018; Zhang et al., 2018). After cultivation for 48 h under 16 h light/8 h dark at 23°C conditions, the injected tobacco plants were treated with 4°C stress for 24 h in an incubator (Dongqi, Ningbo, China). Then the injected tobacco leaves were collected for RNA extraction.

## Results

### High-Throughput Sequencing and Annotation of Peanut sRNAs

To identify endogenous cold stress-responsive miRNAs that are potentially involved in cold response, we treated the two Lines (WQL20 and WQL30) with low-temperature 6°C and sampled tissue from treated plants at four different time points after treatment [0, 4, 8, and 12 h after cold treatment (HCT)]. Total RNA from each sample was used for sRNA library construction. As shown in Table 1, 9,576,091, 8,391,121, 9,343,483, and 7,954,268 total reads were generated from each library representing the different time points in line WQL20, respectively. 9,376,386, 11,205,178, 10,219,016, and 8,626,070 total reads were in line WQL30, respectively. By mapping to the peanut genome (Cultivated peanut, *A*. *hypogaea* cv. Tifrunner),^2^ 9,349,005, 8,234,001, 9,129,232 and 7,784,811 peanut sRNA reads were obtained, corresponding to 2,093,285, 2,057,134, 2,270,139 and 1,634,313 unique reads in WQL20, respectively. 9,242,332, 11,025,444, 9,875,961 and 8,492,492 peanut sRNA reads were obtained in WQL30, corresponding to 1,833,592, 2,107,138, 2,030,541 and 1,998,859 unique reads, respectively. These reads were further searched against the Rfam database^3^ to remove known sRNA, such as ribosomal RNA (rRNA), transfer RNA (tRNA), and small nucleolar RNAs (snoRNAs), the Repbase databases^4^ to remove repeats. In the end, 5,676,589, 5,327,270, 5,435,094 and 4,049,334 reads were retrieved from each library of WQL20, and 4,964,372, 5,982,719, 5,551,346 and 5,019,867 reads were retrieved from each library of WQL30. The reads were considered as sRNA originated.

**Table 1:** Distribution of sRNAs among different categories in each library.

Types	Normalized Reads							
	WQL20				WQL30			
	0 h	4 h	8 h	12 h	0 h	4 h	8 h	12 h
**Total sRNA**	9,576,091	8,391,121	9,343,483	7,954,268	9,376,386	11,205,178	10,219,016	8,626,070
**Mapped sRNA**	9,349,005	8,234,001	9,129,232	7,784,811	9,242,332	11,025,444	9,875,961	8,492,492
Unique reads	2,093,285	2,057,134	2,270,139	1,634,313	1,833,592	2,107,138	2,030,541	1,998,859
**Rfam**								
rRNA	3,024,554	2,104,932	2,902,873	2,898,576	3,674,164	4,202,996	3,637,792	2,867,982
tRNA	144,762	152,863	124,882	215,951	154,697	292,961	187,397	146,373
snRNA	918	844	1,586	873	861	1,584	847	829
snoRNA	13,669	15,491	38,404	16,492	16,673	24,703	13,390	13,998
**Repeat**	488,513	632,601	626,393	603,585	431,565	520,481	485,189	443,443
**Peanut small RNA**	5,676,589	5,327,270	5,435,094	4,049,334	4,964,372	5,982,719	5,551,346	5,019,867
Conserved_miRNA (Total)	1,089,490	942,979	359,409	555,137	1,004,326	1,207,299	1,090,170	881,849
Conserved_miRNA (Unique)	1,328	1,260	1,126	1,184	1,295	1,414	1,339	1,233
Novel_miRNA (Total)	58,424	70,267	34,750	42,150	65,449	103,589	84,218	73,171
Novel_miRNA (Unique)	1,243	1,439	1,398	1,166	1,370	1,546	1,536	1,386
TAS (Total)	3,094	6,961	4,133	2,944	3,508	5,193	5,104	4,408
TAS (Unique)	300	366	298	267	292	333	297	304
NAT (Total)	654,060	757,250	904,777	694,258	687,859	852,495	774,593	663,985
NAT (Unique)	157,239	181,283	199,877	147,463	153,205	180,847	169,278	161,026
exon:+	206,065	230,953	266,696	164,444	183,927	223,993	201,463	170,121
exon:−	126,392	148,788	134,160	99,348	120,449	169,910	139,523	118,291
intron:+	172,157	214,436	249,986	156,912	171,819	209,054	203,835	188,352
intron:−	103,208	127,995	154,537	96,073	109,887	123,634	124,446	120,489
other	3,263,699	2,827,641	3,326,646	2,238,068	2,617,148	3,087,552	2,927,994	2,799,201

*A peanut sRNA deep sequencing summary is presented. The abundance in different libraries was normalized to that in the library at 0 h, and sRNAs were calculated as reads per million*.

The lengths of the unique, valid reads ranged from 18 to 30 nucleotides (nt), and the 21–24 nt sequences were predominant in each library, with the 24 (nt) sequences being the most common (Supplementary Figure S1).

### Identification of Known, Conserved, and Novel miRNAs

To identify known, conserved, and novel miRNAs in peanut, all the total peanut sRNAs were classified into three categories according to their origination and structural features: (1) conserved miRNAs with a hairpin structure of their precursor sequences were aligned to plant miRNAs in miRBase^5^; (2) novel peanut miRNAs have a hairpin structure of their precursor sequences but are not listed in the microRNA database; and (3) peanut siRNAs that do not have a hairpin structure of their precursor sequences. According to this criteria, we retrieved 1,328, 1,260, 1,126, and 1,184 unique conserved peanut miRNAs in each library of WQL20 and 1,295, 1,414, 1,339, and 1,233 unique conserved peanut miRNAs in each library of WQL30, respectively (Table 1; Supplementary Table S1). In addition, we obtained 1,243, 1,439, 1,398, and 1,166 novel peanut miRNAs in each library of WQL20 and 1,370, 1,546, 1,536, and 1,386 novel peanut miRNAs in each library of WQL30, respectively.

### Comparison of Differentially Expressed miRNAs Between the Two Peanut RILs

**Figure 2:**
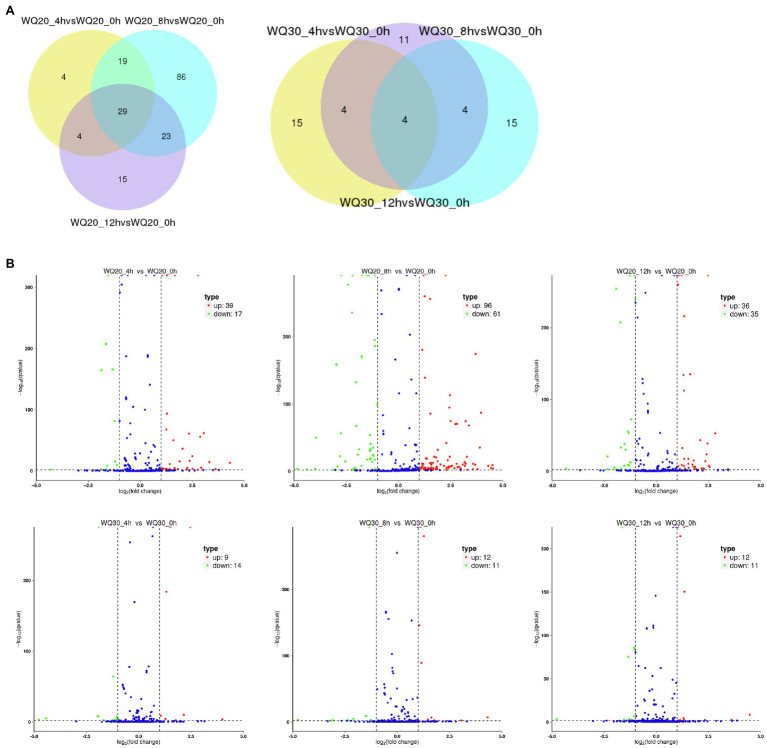
Differentially expressed miRNAs in two peanut RILs. **(A)** Venn diagrams showing the number of common and specific miRNAs in comparisons of the eight libraries. **(B)** Volcanic diagrams showing the number of differentially expressed miRNAs in each comparison. The red dots indicate miRNAs with significant differences, and the blue dots indicate that the difference was not significant for miRNA expression.

To identify the differentially expressed miRNAs, we compared the frequencies of occurrence of differentially expressed miRNAs at the 0 HCT, 4 HCT, 8 HCT, and 12 HCT stages between the two lines after the miRNA reads were normalized to transcripts per million (TPM). We identified 56, 71, and 157 differentially expressed miRNAs in the WQL20_4 h vs. WQL20_0 h, WQL20_8 h vs. WQL20_0 h, and WQL20_12 h vs. WQL20_0 h comparisons, and 23, 23, and 23 differentially expressed miRNAs in the WQL30_4 h vs. WQL30_0 h, WQL30_8 h vs. WQL30_0 h and WQL30_12 h vs. WQL30_0 h comparisons, respectively (Figure 2A). Among these, 39, 96, and 36 miRNAs were upregulated expression, and 17, 61, and 35 miRNAs were downregulated at 4 HDT, 8 HDT, and 12 HDT compared to 0 HDT in RIL WQL20, respectively. 9, 12, and 12 miRNAs were upregulated expression, and 14, 11, and 11 miRNAs downregulated expression in RIL WQL30, respectively (Figure 2B). To identify the miRNAs responsive to cold stress, the miRNAs with opposite changes between the two peanut RILs both abundantly and significantly differentially expressed were identified by employing the following criteria: (1) total reads ≥3,000; (2) [treated/mock] ≥ 2 or [treated/mock] ≤ 0.5 in at least one stage. In the end, we found 12 significantly differentially expressed conserved peanut miRNAs (DE-miRNAs; Table 2).

**Table 2:** List of some candidate miRNAs involved in low-temperature response in each library.

miRNA Identifier		Normalized reads (reads/mgs)								Fold change					
		WQL20				WQL30				WQL20			WQL30		
		0 h	4 h	8 h	12 h	0 h	4 h	8 h	12 h	4 h/0 h	8 h/0 h	12 h/0 h	4 h/0 h	8 h/0 h	12 h/0 h
ahy-miR396	ath-miR396a-5p	12078.55	17044.83	18074.27	27523.55	16443.92	14907.85	14650.86	13752.88	1.41	1.50	2.28	0.91	0.89	0.84
	gma-miR396h	12091.90	17038.83	18086.71	27498.87	16440.42	14907.85	14641.29	13748.15	1.41	1.50	2.27	0.91	0.89	0.84
	zma-miR396g-3p	12058.79	17032.24	18064.93	27494.12	16426.41	14890.62	14634.91	13732.61	1.41	1.50	2.28	0.91	0.89	0.84
	vvi-miR396a	4968.34	6500.29	7497.62	10354.21	3998.15	2943.47	2942.41	2629.60	1.31	1.51	2.08	0.74	0.74	0.66
ahy-miR167	sly-miR167b-5p	7017.83	16039.02	18027.59	18560.48	17306.84	16602.84	19235.74	16172.98	2.29	2.57	2.64	0.96	1.11	0.93
ahy-miR1511	gma-miR1511	2425.43	8460.94	17159.43	15457.25	10430.93	10406.25	8992.64	9098.53	3.49	7.07	6.37	1.00	0.86	0.87
ahy-miR482	gso-miR482a	10571.60	3956.68	7737.22	6511.26	8098.48	10688.83	9343.68	8679.52	0.37	0.73	0.62	1.32	1.15	1.07
ahy-miR2118	pvu-miR2118	1911.19	576.38	692.35	950.53	1144.33	6018.87	2322.76	2762.74	0.30	0.36	0.50	5.26	2.03	2.41
	gma-miR2118a-3p	1911.19	576.38	692.35	950.53	1143.75	6018.87	2322.76	2762.74	0.30	0.36	0.50	5.26	2.03	2.42
ahy-miR156	mdm-miR156ad	978.82	758.11	356.29	997.06	743.82	1090.94	1330.25	1290.14	0.77	0.36	1.02	1.47	1.79	1.73
ahy-miR162	bdi-miR162	656.29	915.25	1754.99	1099.61	629.97	554.33	477.10	408.19	1.39	2.67	1.68	0.88	0.76	0.65
ahy-miR160	ath-miR160a-5p	300.11	267.50	231.82	147.18	280.24	338.21	379.24	339.26	0.89	0.77	0.49	1.21	1.35	1.21

*Variations in expression were analyzed. Peanut miRNAs with opposite changes in WQL20 and WQL30 and significantly differentially expressed were identified using the following criteria: [1] total reads ≥ 3,000; [2] [treated/mock] ≥ 2 or [treated/mock] ≤ 0.5 in at least one stage*.

### Validation of Cold-Responsive miRNAs by Northern Blotting

**Figure 3:**
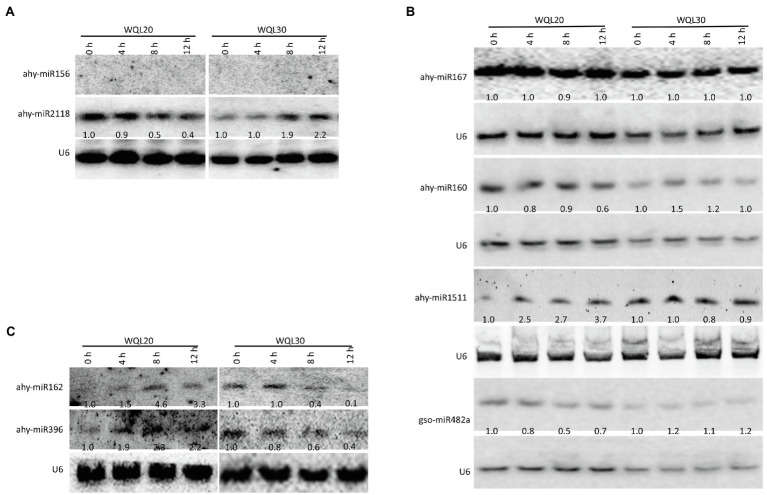
Expression patterns of miRNAs upon low-temperature treatment. Four-leaf stage seedlings were treated with low temperature (6°C), and total RNA was extracted from leaves at the indicated time points. Detection of the peanut miRNAs using Northern blotting. A total of 100 μg of total RNA was loaded. RNA blots were hybridized with DNA oligonucleotide probes complementary to the indicated miRNAs. U6 was used as a loading control. Values below each section represent the relative abundance of miRNA normalized to U6.

By using reverse-cDNA fragments as probes, we validated our bioinformatics prediction by northern blotting assay (there is only one nucleotide difference between ath-miR396a-5p and gma-miR396h, between ath-miR396a-5p and vvi-miR396a, between pvu-miR2118 and gma-miR2118a-3p; therefore, detection using probes reverse complementary to the aforementioned sRNAs represents the expression of the family). The eight northern blottings that were used to detect the candidate miRNAs. ahy-miR2118 and ahy-miR160 showed discernible downregulation in line WQL20 and upregulation in line WQL30 (Figures 3A,B). ahy-miR482 showed discernible downregulation in line WQL20 and weak upregulation in line WQL30 (Figure 3B). ahy-miR1511 showed discernible upregulation in line WQL20 and weak downregulation in line WQL30 (Figure 3B). ahy-miR396 and ahy-miR162 showed discernible upregulation in line WQL20 and downregulation in line WQL30 (Figure 3C). Surprisingly, ahy-miR167 showed strong expression with no noticeable expression variation, which is different to the bioinformatic prediction (Figure 3B). ahy-miR156 expressed under the detectable level (Figure 3A).

### Identification of Target Genes of miRNAs by Degradome Analysis

To further understand the biological functions of cold-responsive miRNAs, the miRNA target genes were identified by Degradome Analysis. In this study, we constructed two degradome libraries, DS1 (including WQL20_0h, WQL20_4h, WQL20_8h, and WQL20_12h) and DS2 (including WQL30_0h, WQL30_4h, WQL30_8h, and WQL30_12h). In total, we obtained 34,299,289 and 43,309,050 raw reads from DS1 and DS2, respectively. After removing the reads without the 3′ adaptor sequence, we obtained 10,139,640 and 9,850,766 unique raw reads from the DS1 and DS2 libraries, respectively. The unique reads were aligned to the peanut genome database, and 25,396,751 (74.04%) and 30,399,096 (70.19%) reads were mapped to the genome, respectively. The mapped reads represented 7,005,430 (69.09%) and 6,538,802 (66.38%) annotated peanut genes in the DS1 and DS2 libraries, respectively (Supplementary Table S2). We identified cleaved targets for miRNAs based on a method in the Cleaveland pipeline (Addo-Quaye et al., 2009), in which a host gene with an alignment score of 7 or less was considered to be a potential target. According to the signature number and abundance of putative cleaved position at each occupied transcript, these cleaved transcripts could be categorized into five classes (0, 1, 2, 3, and 4). In total, 7,561 and 7,296 targets were identified from the DS1 and DS2 libraries, respectively (Supplementary Table S3). For these targets, 291, 70, 2,421, 991, and 3,788 were classified to categories 0, 1, 2, 3, and 4 in the DS1 library, and 365, 41, 2,358, 1,188, and 3,344 belonged to a category <4 in the DS2 library (Supplementary Table S3). There were 11,615 differentially expressed target genes between the DS1 and DS2 libraries. 6,317 genes were upregulated expression for these targets, and 5,298 genes were downregulated expression (Supplementary Table S3).

**Figure 4:**
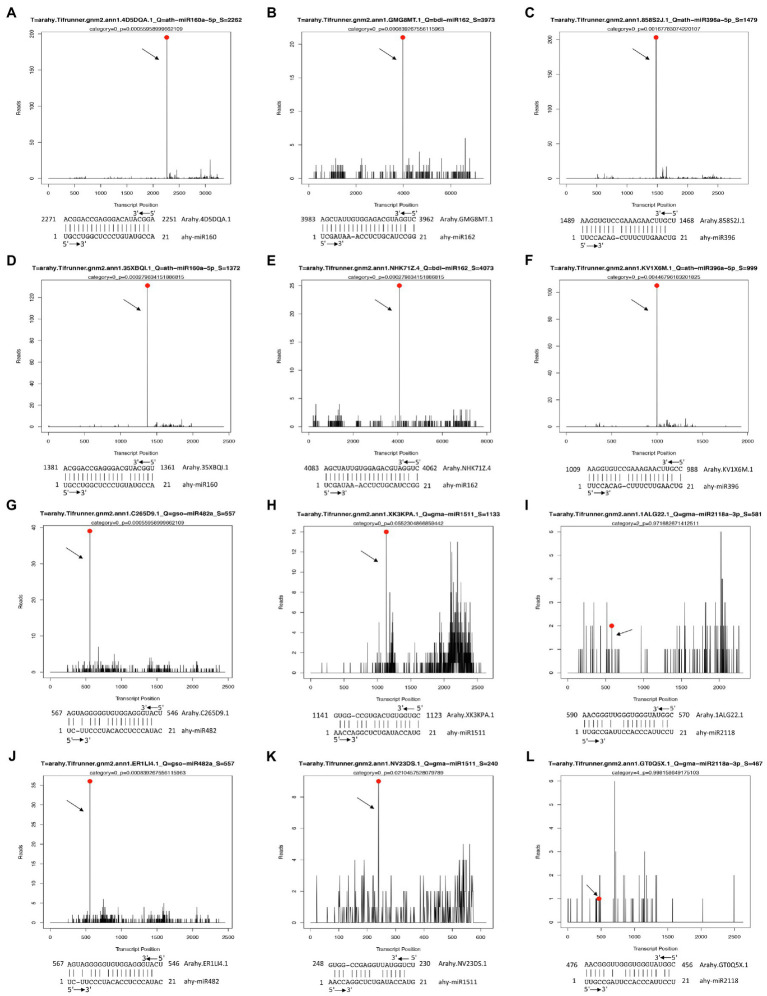
Examples of T-plots of miRNA targets in two peanut RILs confirmed by degradome sequencing. The T-plots show the distribution of the degradome tags along the full length of the target mRNA sequence. The vertical red line indicates the cleavage site of each transcript and is also shown by an arrow. **(A,D)** The cleavage features in *AhARF10* (Arahy.4D5DQA.1) and *ARF17* (Arahy.35XBQI.1) mRNA by ahy-miR160 in DS1. **(B,E)** The cleavage features in *AhDCL6* (Arahy.GMG8MT.1) and *AhDCL16* (Arahy.NHK71Z.4) mRNA by ahy-miR162 in DS1. **(C,F)** The cleavage features in *AhGRF1* (Arahy.858S2J.1) and *AhGRF4* (Arahy.KV1X6M.1) mRNA by ahy-miR396 in DS1. **(G,J)** The cleavage features in *AhWDRL1* (Arahy.C265D9.1) and *AhWDRL2* (Arahy.ER1LI4.1) mRNA by ahy-miR482 in DS1. **(H,K)** The cleavage features in *AhSRF* (Arahy.XK3KPA.1) and *AhSP1L1* (Arahy.NV23DS.1) mRNA by ahy-miR1511 in DS1. **(I,L)** The cleavage features in *AhDR1* (Arahy.1ALG22.1) and *AhDR2* (Arahy.GT0Q5X.1) mRNA by ahy-miR2118 in DS1.

For the cold-responsive miRNAs, we found that ahy-miR160 targets auxin response factor genes *AhARF10* (Arahy.4D5DQA) and *AhARF17* (Arahy.35XBQI) (Figures 4A,D); ahy-miR162 targets DEAD-box ATP-dependent RNA helicase genes *AhDCL6* (Arahy.GMG8MT) and *AhDCL16* (Arahy.NHK71Z) (Figures 4B,E); ahy-miR396 targets the growth-regulating factor 1 genes *AhGRF1* (Arahy.858S2J) and *AhGRF4* (Arahy.KV1X6M) (Figures 4C,F); ahy-miR482 targets WD repeat-containing protein 26-like genes *AhWDRL1* (Arahy.C265D9) and *AhWDRL2* (Arahy.ER1LI4) (Figures 4G,J); ahy-miR1511 targets protein kinase superfamily protein gene *AhSRF* (Arahy.XK3KPA) and SPIRAL1-like 1 gene *AhSP1L1* (Arahy.NV23DS) (Figures 4H,K); ahy-miR2118 targets disease resistance protein genes *AhDR1* (Arahy.1ALG22) and *AhDR2* (Arahy.GT0Q5X) (Figures 4I,L).

**Figure 5:**
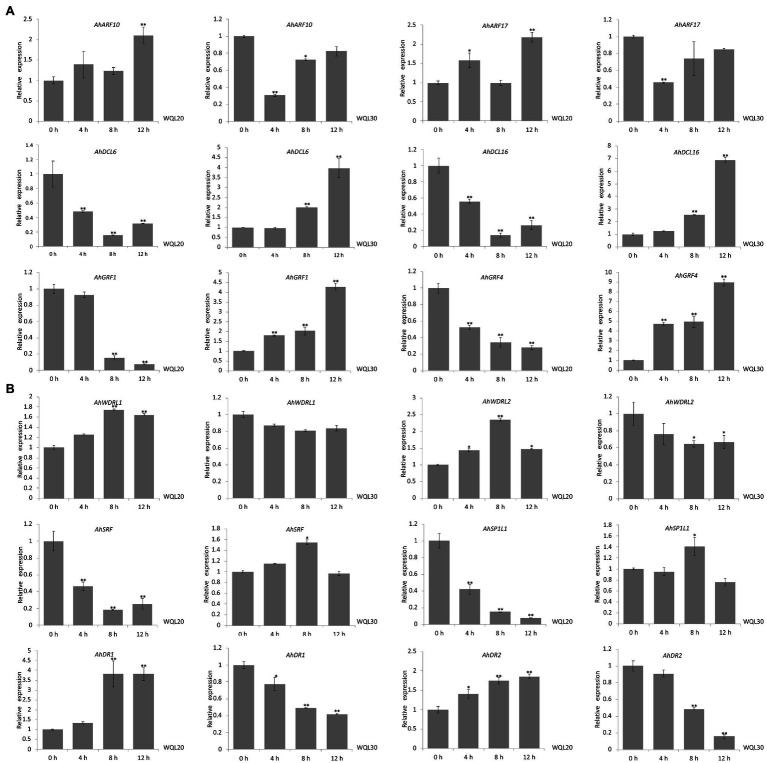
Comparison of the relative expression levels of target genes in two RILs. **(A)** qRT-PCR analyses of target genes of ahy-miR160, ahy-miR162, and ahy-miR396 expression levels in two peanut RILs upon low-temperature (6°C) treatment at the indicated time points, respectively. **(B)** qRT-PCR analyses of target genes of ahy-miR482, ahy-miR1511, and ahy-miR2118 expression levels in two peanut RILs upon low-temperature (6°C) treatment at the indicated time points, respectively. Data are shown as means ± SD (*n* = 3). Student’s *t*-test was used to determine the significance of differences between 0 HCT and the indicated time points. Asterisks indicate significant differences (^*^p < 0.05 and ^**^p < 0.01). Similar results were obtained from three biological replicates.

We further examined the *in vivo* expression of each predicted target gene upon low-temperature treatment. The RNA used for qRT-PCR was from the same set RNA for northern blot analyses. Both of the transcriptions of *ARF10* and *ARF17* (targeted by ahy-miR160) were significantly upregulated expression in line WQL20, but significantly downregulated expression in line WQL30, respectively. Same as *AhGRF1* and *AhGRF4* (targeted by ahy-miR396), *AhDCL6* and *AhDCL16* (targeted by ahy-miR162) showed similar expression profiles between the two RIL lines. In line WQL20, they were significantly downregulated expression, but significantly upregulated expression in line WQL30, respectively (Figure 5A). *AhDR1* and *AhDR2*, target genes of ahy-miR2118, showed sustained increase with the low-temperature treatment in line WQL20, while sustained decrease in line WQL30, respectively. The transcriptions of *AhSRF* and *AhSP1L1* showed a noticeable reduction since 4 h and reached less than 40% of the original level at 8 h in line WQL20, but significantly increase in WQL30 with low-temperature treatment, which corresponds to the overall expression tendency of ahy-miR1511. The expressions of *AhWDRL1* and *AhWDRL2* are cognately inverse to the expression pattern of ahy-miR482, further suggesting a regulatory relationship between these two parties (Figure 5B). In summary, our research indicates that these genes are authentic target genes of their cognate miRNAs. These miRNAs may be involved in the cold-responsive process in peanut by negatively regulating their target genes.

### Functional Analysis of the Six Cold-Responsive miRNAs in Tobacco

**Figure 6:**
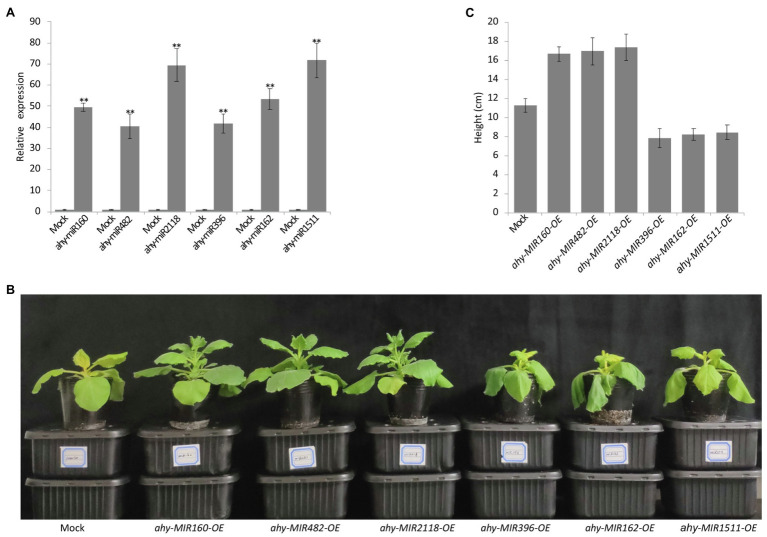
Functional analysis of the six cold-responsive miRNAs in tobacco infiltrated by *Agrobacterium tumefaciens* strain GV3101. **(A)** The transcript analysis of peanut miRNAs in infiltrated tobacco leaves. Student’s *t*-test was used to determine the significance of differences between mock and the indicated lines. Asterisks indicate significant differences (^**^p < 0.01). Similar results were obtained from three biological replicates. **(B)** The phenotype observed in infiltrated tobacco leaves overexpressing pCAMBIA1300 and pCAMBIA1300-*MIRNAs* with 4°C treatment for 24 h. **(C)** The different height of infiltrated tobacco overexpressing pCAMBIA1300 and pCAMBIA1300-*MIRNAs* with 4°C treatment for 24 h.

To further analyze the function of cold-responsive miRNAs, we transiently overexpressed the six cold-responsive miRNAs in tobacco leaves using Agrobacterium expressing pCAMBIA1300-miRNAs (Figure 6A). After 24 h cold treatment, the tobacco plants showed visual phenotypes. Compared with the control, the leaves overexpressing ahy-miR160, ahy-miR482, and ahy-miR2118 exhibited better growth status, but the leaves overexpressing ahy-miR396, ahy-miR162, and ahy-miR1511 showed poor growth status (Figures 6B,C). These results suggest that tobacco leaves overexpressing ahy-miR160, ahy-miR482, and ahy-miR2118 have enhanced cold tolerance. On the contrary, tobacco leaves overexpressing ahy-miR396, ahy-miR162, and ahy-miR1511 have weakened cold tolerance.

## Discussion

### The Cold-Responsive miRNAs in Peanut

Cold stress is the main limiting factor for the distribution, yield, and quality of crops (Zhang et al., 2019). The cold signaling pathway has been well studied (Jeon and Kim, 2013; Barrero-Gil and Salinas, 2018; Shi et al., 2018), and many studies have shown that miRNAs play critical roles in cold stress in plants. For example, osa-miR319 can silence *OsPCF5* and *OsPCF8*, and positively regulate cold tolerance in rice (Yang et al., 2013); osa-miR156k targets the cold stress-related gene *SPL* and negatively regulates cold tolerance in rice (Cui et al., 2015); sha-miR319 can silence *Gamyb*-*like1* genes that positively regulate cold tolerance in tomatoes (Shi et al., 2019). As a typical semi-arid tropical and subtropical plant, peanuts are highly sensitive to cold stress and may have evolved differential regulatory mechanisms to respond to cold stress. However, the function of small RNA-mediated gene silencing in peanut cold defense has not been reported. In this study, the transcription levels of miRNAs were analyzed between tolerant and sensitive lines with cold treatment in peanuts, and 407 known miRNAs and 143 novel peanut-specific miRNAs in total were identified. Our data showed that known miRNAs and novel miRNAs were involved in cold responses from eight libraries (Supplementary Table S1). Some of these miRNAs have been reported to be responsive to low temperatures in other plants. For example, specific members of the miR171 and miR396 families were also found to respond to cold treatment in peanuts, indicating that they are also conserved in dicotyledons. Likewise, some previously identified cold-induced conserved miRNAs, such as miR393 and miR402 in *Arabidopsis* (Sunkar and Zhu, 2004), were not found in our data, suggesting that they were unaffected by cold or that induction levels of certain miRNAs may be too low to be detected as significant in peanuts. Finally, through experimental verification, we found that six miRNAs might play key roles in peanut cold defense.

Based on miRNA expression profiling and degradome analysis combined with northern blotting and qRT-PCR validation, we found that several miRNA target pairs (miR160-*ARF*, miR482-*WDRL*, miR2118-*DR*, miR162-*DCL*, miR1511-*SRF*, miR1511-*SPIRAL1*, and miR396-*GRF*) might play key roles in the regulation of cold tolerance in peanuts. Specially, miR160-*ARF*, miR482-*WDRL,* and miR2118-*DR* showed opposite expression profiles to miR162-*DCL*, miR1511-*SRF*, miR1511-*SPIRAL1*, and miR396-*GRF* in tolerant and sensitive lines, indicating that they may be key factors leading to differences in cold tolerance.

### mirnas Mediated Positive Regulation Pathway in Peanut Cold Response

ahy-miR160, ahy-miR482, and ahy-miR2118 were upregulated in cold-tolerant line WQL30 but downregulated in cold-sensitive line WQL20 (Figure 3). According to previous reports, highly conserved miR160 with ARF targets responds to cold tolerance in maize, wheat, *Populus trichocarpa*, *Populus simonii* × *P. nigra*, and wild banana (Lu et al., 2005; Liu et al., 2018; Zhou et al., 2019; Aydinoglu, 2020). Likewise, the target gene auxin response factor (*ARF*) not only regulates plant growth but also participates in abiotic stress responses, especially cold stress. For example, in *Arabidopsis*, *AtARF* genes are upregulated during cold acclimation (Hannah et al., 2005). Many *ARF* genes are repressed or induced in bananas (*Musa acuminata* L.) following cold, salt, and osmotic stress (Hu et al., 2015). In maize, 13 *ARFs* were upregulated in seedling leaves after cold stress (Aydinoglu, 2020). SLR/IAA14, a transcriptional repressor of auxin signaling, plays a crucial role in the integration of miRNAs in auxin and cold responses in *Arabidopsis* roots (Aslam et al., 2020). In peanuts, the target genes of ahy-miR160 (*AhARF10* and *AhARF17*) also responded to cold stress (Figures 4, 5), and overexpression of ahy-miR160 enhanced cold tolerance in tobacco (Figure 6). These studies indicate that under cold stress conditions, upregulated ahy-miR160 may reduce ARF transcript levels, suppressing *ARF*-mediated auxin-responsive gene expression, leading to peanut growth and development attenuation, eventually enhancing cold stress tolerance in peanuts.

Studies have reported that miR2118 targets the protein family associated with disease resistance in response to biotic and abiotic stresses in many plants (de Vries et al., 2015; Wu et al., 2015; Cakir et al., 2016; Gao et al., 2016; Yang et al., 2020; Shi et al., 2021). In *Caragana intermedia*, cin-miR2118, a drought-resistant miRNA, positively affects drought stress tolerance. Overexpression of cin-miR2118 enhances drought tolerance in tobacco (Wu et al., 2015). miR2118 is a cold-responsive sRNA in wheat, banana, *Astragalus membranaceus*, and cassava (Xia et al., 2014; Song et al., 2017; Abla et al., 2019; Zhu et al., 2019). Similarly, our data confirmed that ahy-miR2118 was also a cold-responsive miRNA (Figure 3), and *DR* genes could also be targeted by ahy-miR2118 *via* the anti-correlation between miR2118 and the corresponding target *AhDR* genes in peanuts (Figures 4, 5). Moreover, enhanced cold tolerance was also observed in tobacco plants overexpressing ahy-miR2118 (Figure 6), indicating that miR2118 might play an important role in plant resistance to abiotic stress. Under cold treatment, pdu-miR482 was differentially expressed between H (a cold-tolerant genotype) and Sh12 (a cold-sensitive genotype) in almonds (Karimi et al., 2016), respectively. Csn-miR482 was also significantly different between two tea plant cultivars, “Yingshuang” (YS, a cold-tolerant tea plant cultivar) and “Baiye 1” (BY, a cold-sensitive tea plant cultivar; Zhang et al., 2014). In peanuts, the repression in sensitive lines and weak induction in tolerant lines for ahy-miR482 was consistent with the findings of the ovary tissues of H genotype and Sh12 under 0°C treatment in almonds (Figure 3). ahy-miR482 targeted *AhWDRL*s (Figure 4). *AhWDRL*s belong to the WD40 protein family, and the large gene family of WD40 proteins is involved in a broad spectrum of crucial plant stress resistance processes. HOS15, a WD40-repeat protein, plays a role in gene activation/repression *via* histone modification during plant acclimation to low-temperature conditions (Sharma and Pandey, 2015). RNA-seq data analysis also showed that numerous *TaWD40s* are involved in responses to cold stress in wheat (Hu et al., 2018). Similarly, we found that *AhWDRL* genes are also involved in responses to cold stress in peanuts (Figure 5). Moreover, the phenomenon of enhanced cold tolerance was observed in tobacco by overexpressing ahy-miR482 (Figure 6). In brief, miR160-*ARF*, miR482-*WDRL*, and miR2118-*DR* modules might mediate cold stress responses, and these three miRNAs may play roles in the positive regulation of peanut cold tolerance through related target genes in peanuts. Therefore, our data sheds light on the possibility of manipulating miRNAs to improve the tolerance of peanuts to cold stress.

### miRNAs Mediated Negative Regulation Pathway in Peanut Cold Response

In addition to the above-mentioned miRNAs, we found three other miRNAs with differential responses to cold stress in tolerant and sensitive lines: ahy-miR396, ahy-miR162, and ahy-miR1511, which were upregulated in the cold-sensitive line WQL20 but downregulated in the cold-tolerant line WQL30 (Figure 3). Overexpressing the three miRNAs weakened cold tolerance in tobacco, which means that they may play roles as negative regulators of cold tolerance in peanuts.

Previous studies have shown that miR396 mediates the silencing of target gene *GRFs* and participates in the regulation of the cold signal response in many plants, such as *Arabidopsis thaliana*, rice, soybean, tomato, poplar, wheat, and wild grapes (Lu et al., 2008; Tang et al., 2012; Zhang et al., 2014; Yan et al., 2016; Chen et al., 2019; Shi et al., 2019; Wang et al., 2019; Lantzouni et al., 2020). GRFs are a family of transcription factors that mediate the development of seeds, leaves, flowers, root growth, and other essential life processes in plants, such as *Arabidopsis thaliana*, *Brassica napus*, *Glycine max* (soybean), *Solanum tuberosum* (potato), *Oryza sativa* (rice), and *Zea mays* (maize) (Kim et al., 2003; Omidbakhshfard et al., 2015). Additionally, GRFs are also the critical factors of cold signal transduction in plants, which can directly bind to the promoter region of the *CBF* gene to inhibit its transcription. For example, *Arabidopsis AtGRF7* can bind to the tgtcagg cis element on the *AtCBF3* promoter to inhibit the expression of *AtCBF3* at the transcriptional level (Kim et al., 2012). Lantzouni et al. (2020) found that *AtCBF* genes (*AtCBF*1, *AtCBF*2, and *AtCBF*3) and the downstream *AtCOR* genes (*AtCOR*15a, *AtCOR15b*, and *AtCOR29d*) were significantly downregulated *in vivo* after overexpression of *GRF5*, and miR396/*GRF* module is a vital regulation mode for plants to balance growth, development, and cold response under cold stress. In peanuts, we also observed that ahy-miR396 can target *AhGRFs* and that overexpression of ahy-miR396 weakened cold tolerance in tobacco (Figures 4, 6), suggesting that it might help alleviate cold tolerance in peanuts through the target *GRF* gene. However, there is no direct evidence for a relationship between *AhGRFs* and the CBF pathway in peanuts.

Studies have shown that miR1511also is a cold-responsive sRNA in many plants. For example, pdu-miR1511 was differentially expressed between H and Sh12 in almond under cold treatment (Karimi et al., 2016). csi-miR1511 was also significantly different between YS and BY in tea plant (Zhang et al., 2014). mit-miR1511 showed remarkably higher levels at the cold stress temperatures (0°C) in the wild bananas (Liu et al., 2018). In agreement with previous reports, ahy-miR1511 showed significant differences between the tolerant and sensitive lines (Figure 3). It targets *the AhSRF* and *AhSPIRAL1* genes (Figures 4, 5). In *Arabidopsis*, *AtSPIRAL1* maintains microtubules (MT) stability and is closely associated with stress. For example, salt stress can lead to the UPS-dependent degradation of the MT-associated protein AtSPIRAL1. The degradation of AtSPIRAL1 results in the depolymerization of MTs, followed by the formation of new MTs that are better adapted to osmotic stress (Wang et al., 2011). For another target gene *AhSRF*, recent evidence has shown that homologous gene *AtSRF6* is an identified regulator for *COR* in *Arabidopsis* (Wei et al., 2021). These reports suggest that the miR1511-*SRF* and miR1511-*SPIRAL1* modules may attenuate cold tolerance by mediating MT stability or by regulating the ICE-CBF-COR pathway. Another anti-correlated module in peanuts is ahy-miR162, which targets *AhDCL*. It has been reported that miR162 not only regulates the key factors of plant growth and development but also responds to low-temperature stress. In almonds, pdu-miR162 is upregulated in H and downregulated in Sh12 under cold stress (Karimi et al., 2016). This difference in expression in tolerant and sensitive varieties agreed with our data in peanuts (Figure 3), suggesting that miR162 is conserved and functional in plant cold tolerance. Pdu-miR162 directly targets *PdDCL1* for cleavage (Karimi et al., 2016). DCL proteins are key regulators of small RNA biogenesis (RNA interference, RNAi), and participate in the response to cold stress in plants. For example, rice, like peanuts, are low temperature-sensitive plants. Vyse et al. (2020) found that *OsDCL1* is induced by cold acclimation in rice. We also observed significant differences in the expression of *AhDCL6* and *AhDCL16* targeted by ahy-miR162 in tolerant and sensitive lines (Figures 4, 5), suggesting that the miR162-*DCL* module probably weakens cold stress tolerance in peanuts by feedback regulation of small RNA biogenesis.

## Conclusion

In summary, cold-responsive miRNAs and candidate target genes were identified through integrated sRNA and degradome analysis during cold treatment of tolerant and sensitive lines. ahy-miR160, ahy-miR162, ahy-miR396, ahy-miR482, ahy-miR1511, and ahy-miR2118, as well as transcriptional factors, including GRF, WDRL, and ARF, or genes, such as *SRF*, *DCL*, and *SPIRAL*, were differentially expressed. They might be the main contributors to the CBF and ARF pathways, protein kinase, small RNA biogenesis, and MT stability involved in cold tolerance of peanuts. These miRNAs may downregulate the expression of their target genes, which encode the regulatory and functional proteins involved in cold tolerance. These results increase our knowledge of sRNAs involved in the post-transcriptional regulation of cold tolerance and provide candidate genes for future functional analyses of cold tolerance-related signaling pathways in peanuts.

## Data Availability Statement

The raw data of the sRNA and degradome libraries are available from the NCBI Sequence Read Archive (SRA) under accession numbers SRR19241918, SRR19241919, SRR19241920, SRR19241921, SRR19241922, SRR19241923, SRR19241924, SRR19241925 and SRR19262409, SRR19262410, respectively.

## Author Contributions

DB and HJ designed the experiments. XZ, CR, YX, YT, HZ, NL, and CS performed the experiments. XZ, DB, and HJ performed the data analysis. XZ wrote the manuscript. All authors contributed to the article and approved the submitted version.

## Funding

This work was supported by the National Natural Science Foundation of China (31871662), the Open Project of Key Laboratory of Biology and Genetic Improvement of Oil Crops, Ministry of Agriculture and Rural Affairs, P. R. China (KF2021004), the China Agricultural Research System (CARS-13), the Cultivation Project of National Natural Science Foundation (YGJPY1901), the Doctoral Research Fund Project, Technology Innovation Research Project of the Shanxi Academy of Agricultural Sciences (YBSJJ2014), the Research Program Sponsored by State Key Laboratory of Sustainable Dryland Agriculture (in preparation), Shanxi Agricultural University (NO. 202105D121008), and the earmarked fund for Modern Agro-industry Technology Research System (2022-05).

## Conflict of Interest

The authors declare that the research was conducted in the absence of any commercial or financial relationships that could be construed as a potential conflict of interest.

## Publisher’s Note

All claims expressed in this article are solely those of the authors and do not necessarily represent those of their affiliated organizations, or those of the publisher, the editors and the reviewers. Any product that may be evaluated in this article, or claim that may be made by its manufacturer, is not guaranteed or endorsed by the publisher.

## Footnotes

^1^ http://www.peanutbase.org

^2^ http://www.peanutbase.org

^3^ http://xfam.org/

^4^ http://www.girinst.org/repbase/update/index.html

^5^ http://www.mirbase.org/

## Supplementary Material

Supplementary Figure S1 Length distribution and abundance of the sequences. The lengths of the unique, valid reads ranged from 18 to 30 nucleotides (nt), and the 21-24 nt sequences were predominant in all libraries, with the 24 (nt) sequences being the most common.

Supplementary Figure S2 Examples of T-plots of miRNA targets in two peanut RILs confirmed by degradome sequencing. The T-plots show the distribution of the degradome tags along the full length of the target mRNA sequence. The vertical red line indicates the cleavage site of each transcript and is also shown by an arrow. **(A,D)** The cleavage features in *AhARF10* (Arahy.4D5DQA.1) and *AhARF17* (Arahy.35XBQI.1) mRNA by ahy-miR160 in DS2. **(B,E)** The cleavage features in *AhDCL6* (Arahy.GMG8MT.1) and *AhDCL16* (Arahy.NHK71Z.4) mRNA by ahy-miR162 in DS2. **(C,F)** The cleavage features in *AhGRF1* (Arahy.858S2J.1) and *AhGRF4* (Arahy.KV1X6M.1) mRNA by ahy-miR396 in DS2.

Supplementary Figure S3 Examples of T-plots of miRNA targets in two peanut RILs confirmed by degradome sequencing. The T-plots show the distribution of the degradome tags along the full length of the target mRNA sequence. The vertical red line indicates the cleavage site of each transcript and is also shown by an arrow. **(A,D)** The cleavage features in *AhWDRL1* (Arahy.C265D9.1) and *AhWDRL2* (Arahy.ER1LI4.1) mRNA by ahy-miR482 in DS2. **(B,E)** The cleavage features in *AhSRF* (Arahy.XK3KPA.1) and *AhSP1L1* (Arahy.NV23DS.1) mRNA by ahy-miR1511 in DS2. **(C,F)** The cleavage features in *AhDR1* (Arahy.1ALG22.1) and *AhDR2* (Arahy.GT0Q5X.1) mRNA by ahy-miR2118 in DS2.

Supplementary Table S1 List of identified miRNAs differentially expressed in peanut with cold treatment.

Supplementary Table S2 Statistics of degradome reads in DS1 and DS2.

Supplementary Table S3 Differentially-expressed target genes between the DS1 and DS2 libraries.

Supplementary Table S4 Primer information.
