# Supplementary figures and images for: Small RNA and Degradome Deep Sequencing Reveals the Roles of microRNAs in Peanut (Arachis hypogaea L.) Cold Response
Source: Front Plant Sci. 2022 Jun 2;13:920195. doi: 10.3389/fpls.2022.920195 (PMC9203150; doi:10.3389/fpls.2022.920195)

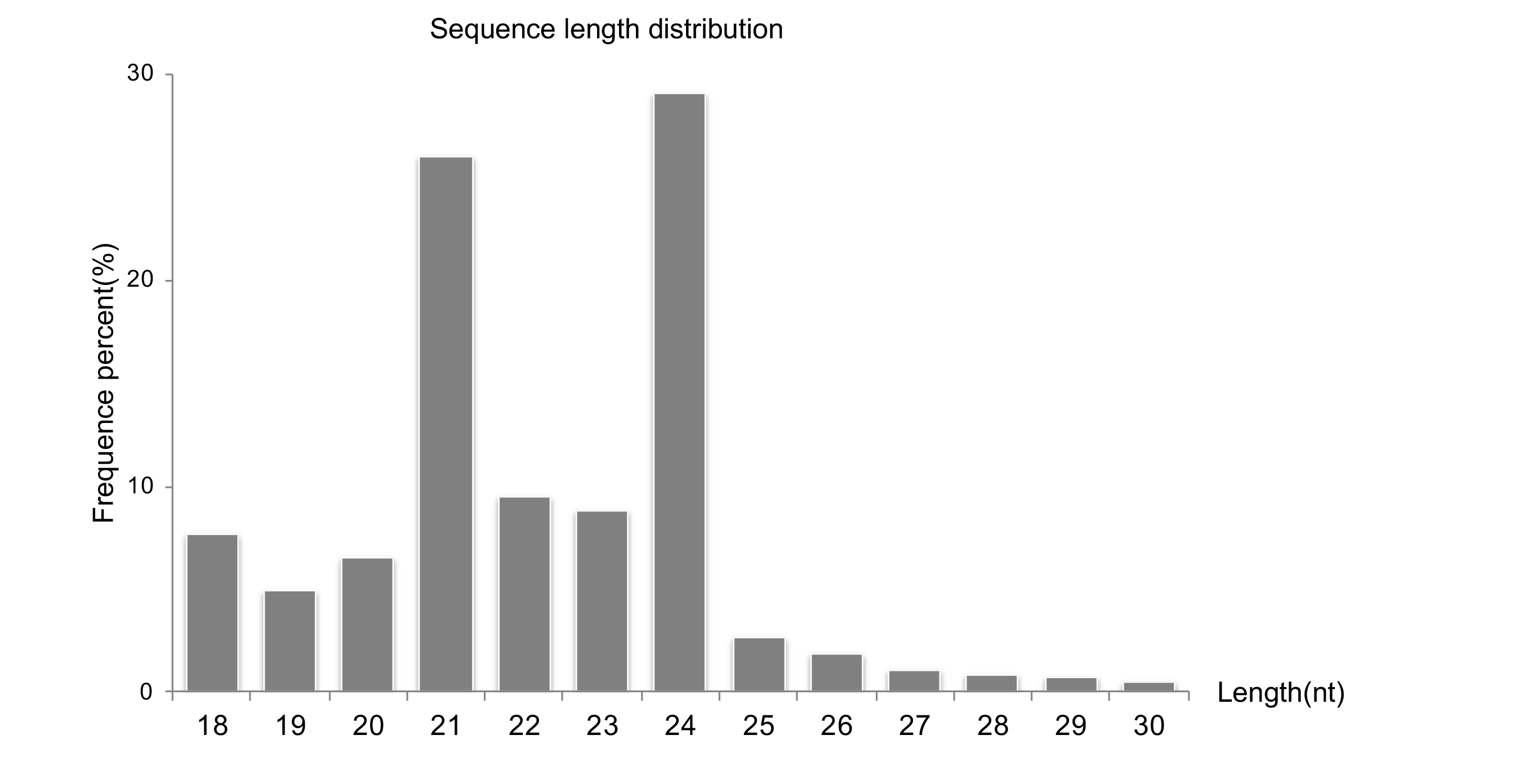

Supplement: Supplementary Figure S1 — Length distribution and abundance of the sequences. The lengths of the unique, valid reads ranged from 18 to 30 nucleotides (nt), and the 21-24 nt sequences were predominant in all libraries, with the 24 (nt) sequences being the most common. [file Image_1.jpg]

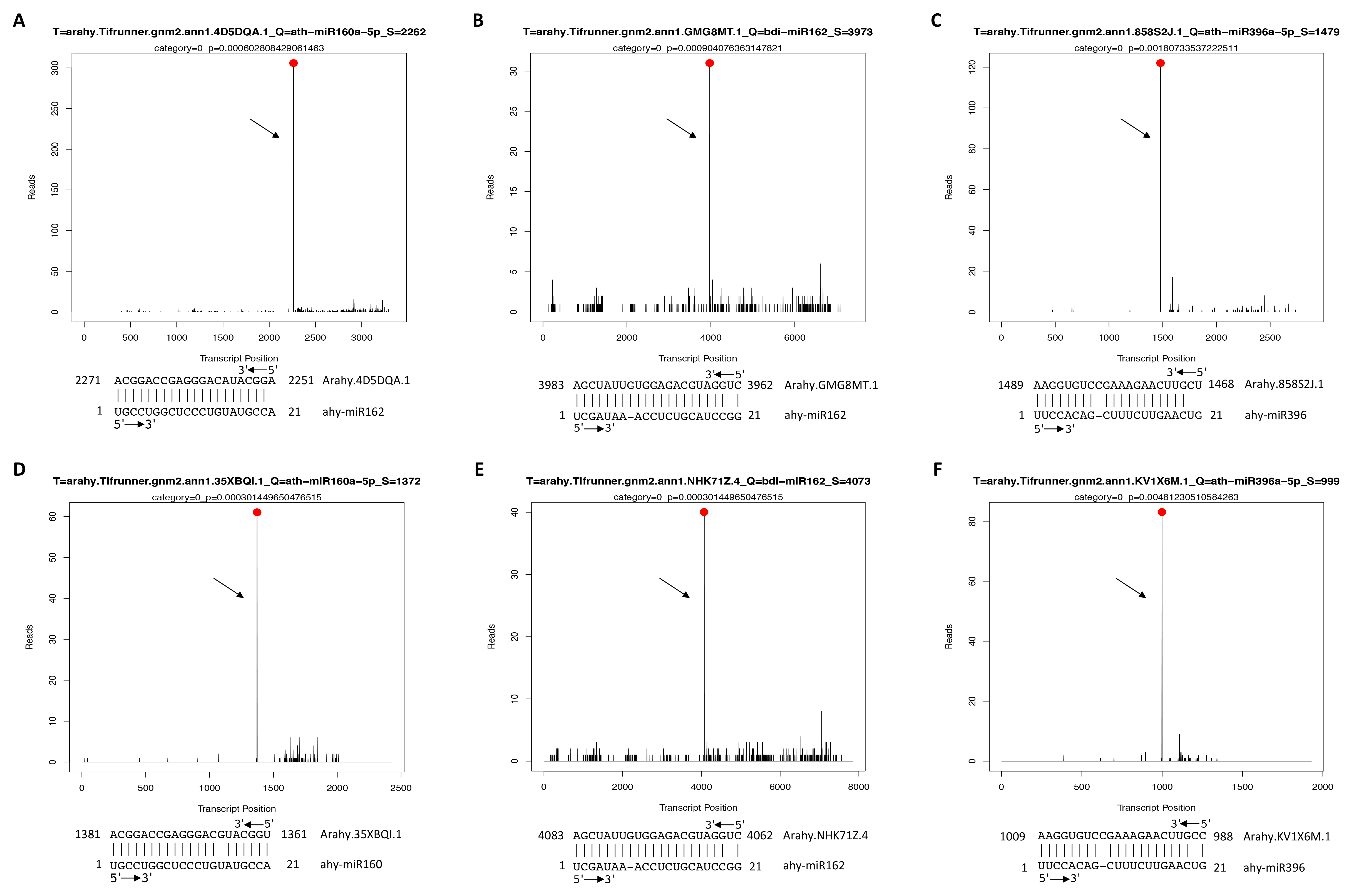

Supplement: Supplementary Figure S2 — Examples of T-plots of miRNA targets in two peanut RILs confirmed by degradome sequencing. The T-plots show the distribution of the degradome tags along the full length of the target mRNA sequence. The vertical red line indicates the cleavage site of each transcript and is also shown by an arrow. (A,D) The cleavage features in AhARF10 (Arahy.4D5DQA.1) and AhARF17 (Arahy.35XBQI.1) mRNA by ahy-miR160 in DS2. (B,E) The cleavage features in AhDCL6 (Arahy.GMG8MT.1) and AhDCL16 (Arahy.NHK71Z.4) mRNA by ahy-miR162 in DS2. (C,F) The cleavage features in AhGRF1 (Arahy.858S2J.1) and AhGRF4 (Arahy.KV1X6M.1) mRNA by ahy-miR396 in DS2. [file Image_2.jpg]

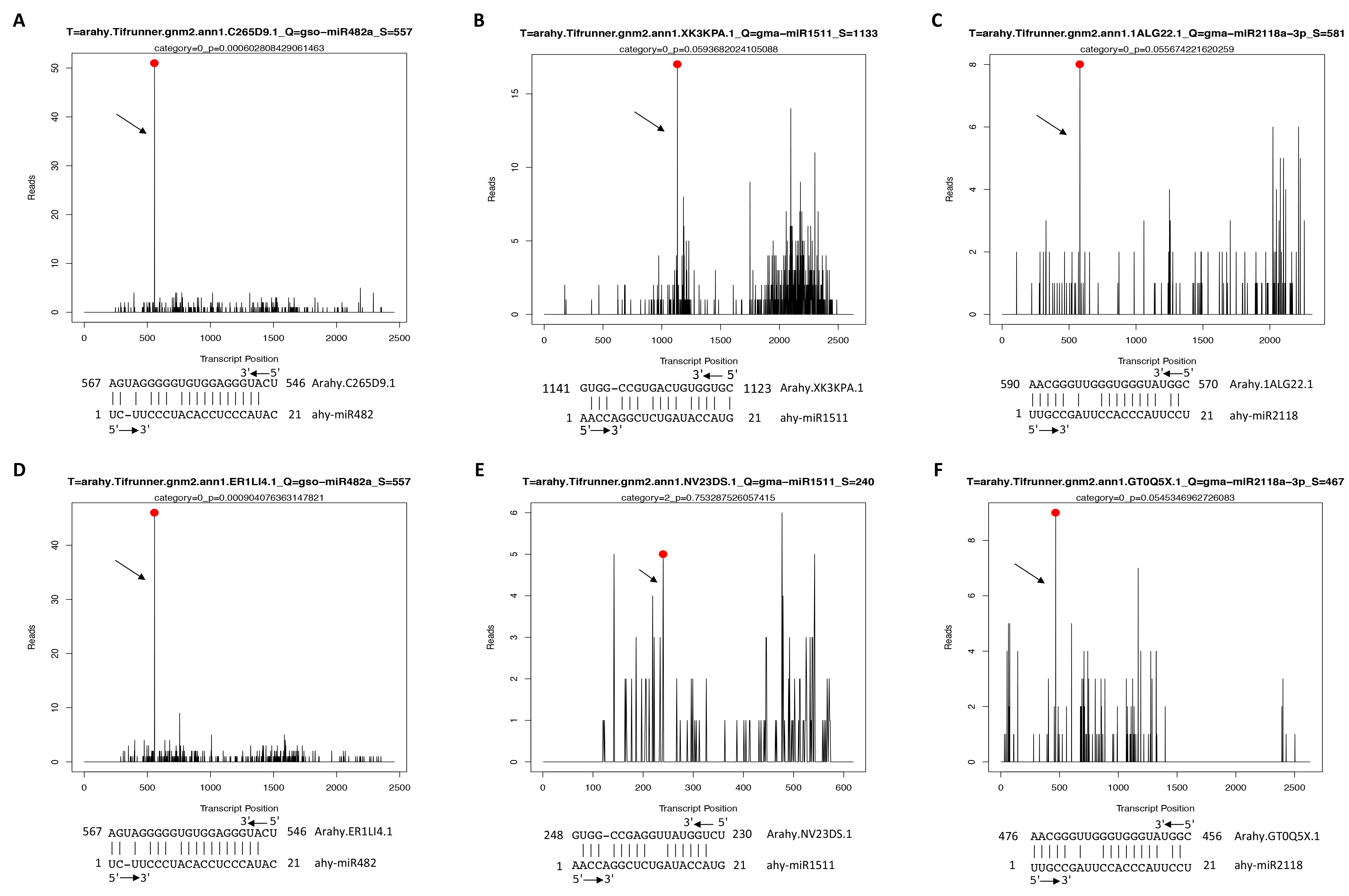

Supplement: Supplementary Figure S3 — Examples of T-plots of miRNA targets in two peanut RILs confirmed by degradome sequencing. The T-plots show the distribution of the degradome tags along the full length of the target mRNA sequence. The vertical red line indicates the cleavage site of each transcript and is also shown by an arrow. (A,D) The cleavage features in AhWDRL1 (Arahy.C265D9.1) and AhWDRL2 (Arahy.ER1LI4.1) mRNA by ahy-miR482 in DS2. (B,E) The cleavage features in AhSRF (Arahy.XK3KPA.1) and AhSP1L1 (Arahy.NV23DS.1) mRNA by ahy-miR1511 in DS2. (C,F) The cleavage features in AhDR1 (Arahy.1ALG22.1) and AhDR2 (Arahy.GT0Q5X.1) mRNA by ahy-miR2118 in DS2. [file Image_3.jpg]
